# Supreme nasal conchae in human cadavers: prevalence, morphometry, and a new classification proposal

**DOI:** 10.1007/s00276-026-03902-1

**Published:** 2026-05-28

**Authors:** Mauro Bezerra Montello, Sofia Helyeth Ramirez Cardenas, Themístocles da Silva Negreiros Neto, Gabriel Vitório de Araújo Suassuna, Wigínio Gabriel Lira-Bandeira, Ingrid C. Landfald, Łukasz Olewnik, Judney Cley Cavalcante, Bento João Abreu

**Affiliations:** 1 Group of Study and Research in Human Anatomy (NEPAH), Laboratory of Human Anatomy, Department of Morphology, Federal University of Rio Grande Do Norte, Universitary Campus, Av Salgado Filho S/N, Lagoa Nova, Natal, RN 59078-900 Brazil; 2 Department of Clinical Anatomy, Mazovian Academy in Płock, Płock, Poland; 3 VARIANTIS Research Laboratory, Department of Clinical Anatomy, Mazovian Academy in Płock, Płock, Poland

**Keywords:** Supreme nasal concha, Zuckerkandl’s concha, Nasal turbinates, Ethmoid bone, Endoscopic sinus surgery, Principal component analysis

## Abstract

**Background:**

Extranumerary nasal conchae, including the supreme nasal concha (SupNC) and Zuckerkandl’s concha (ZC), are uncommon variants relevant to endoscopic sinus and skull base surgery, yet their prevalence and morphometry remain poorly defined. No cadaveric study has simultaneously evaluated prevalence, detailed morphometry, and a data-driven SupNC classification using principal component analysis (PCA). This study determined SupNC prevalence in cadavers and proposed a clinically oriented classification integrating morphological and quantitative parameters.

**Material and methods:**

A cross-sectional cadaveric study analyzed 59 hemisections with preserved nasal conchae. Conchae were counted, and SupNCs classified according to Orhan and a new system. Morphometry was obtained using digital calipers and ImageJ.

**Results:**

Of 59 hemisections, 37 (63%) had three conchae, 21 (35%) four, and 1 (2%) five. SupNC was present in 22/59 (37.3%) and ZC in 1/59 (1.7%), associated with SupNC. Significant differences were found between hemisections with and without extranumerary conchae in middle concha area (3.68 ± 1.07 vs. 4.45 ± 1.20 mm^2^), superior concha area (1.26 ± 0.40 vs. 0.94 ± 0.36 mm^2^), length (26.13 ± 4.19 vs. 21.93 ± 3.94 mm), and height (0.72 ± 0.24 vs. 0.45 ± 0.13 mm). Concha number strongly correlated negatively with nasal cavity height (r = –0.83). PCA identified two clusters: Straight (Type I) and Triangular (Type II) SupNCs.

**Conclusion:**

SupNC was relatively common, whereas ZC was rare. The proposed morphology- and morphometry-based classification may improve preoperative CT evaluation and intraoperative orientation.

## Introduction

The lateral wall of the nasal cavity comprises three nasal conchae: the superior nasal concha (SNC) and middle nasal concha (MNC), which are parts of the ethmoid bone; and the inferior nasal concha (INC), an independent bone [19]. Also known as turbinates, each concha consists of a thin bony lamina covered by tightly adherent mucoperiosteum, which markedly increases the intranasal surface area, thereby enhancing air humidification and warming [7].

Clinically, the nasal conchae serve as important anatomical landmarks for the safe performance of endoscopic sinus surgery [10]. In addition, anatomical variations of the conchae have been associated with a range of symptoms, including nasal obstruction [17], sinusitis [20], and headache [22]. In cases of asymmetric structural development, these variations may also lead to alterations in mucociliary transport and impaired function of the paranasal sinuses [16].

In addition to the three main conchae and their variations, two further anatomical variants have been described in the literature [4]. The fourth concha, also known as the supreme nasal concha (SupNC), was first reported by Santorini in 1724 [13] and has a reported prevalence ranging from 8 to 77% [6]. The fifth concha (Zuckerkandl’s concha, ZC), described by Zuckerkandl in 1882, has no documented prevalence and is generally regarded as a rare finding [13]. Both extranumerary conchae are projections of the ethmoid bone located in the superior region of the nasal cavity, and they may occasionally fuse posteriorly through shared extensions [21]. In this arrangement, they are positioned from inferior to superior as follows: the SNC, the SupNC, and the ZC [21].

Despite increasing evidence, morphometric and prevalence data on the SupNC remain scarce, particularly from cadaveric series with systematic measurements. Most available studies have relied on imaging examinations, including computed tomography (CT) [6], which may be less sensitive than direct cadaveric analysis for detecting small accessory nasal conchae, and on case reports or small cadaveric samples without comprehensive morphometry [3, 21]. Data on ZC are even more limited and largely confined to isolated case descriptions, precluding any robust estimate of its prevalence [21]. The three main nasal conchae are known to exhibit frequent anatomical variations in both their characteristics [14] and shape [5, 23], yet comparable, quantitative information on the SupNC and other accessory conchae is lacking, despite recognition of their distinct morphological forms. Orhan et al. [18] proposed a length-based classification of the SupNC relative to the SNC (shorter, equal or longer), but this system does not capture the full spectrum of morphological patterns and relationships to the superior meatus. Consequently, there is still no cadaveric study that integrates prevalence, detailed morphometry and data-driven clustering, for example via principal component analysis (PCA), into a clinically oriented classification framework for the SupNC.

Therefore, the aims of this study were to: (i) determine the prevalence of the SupNC and ZC in human cadavers; (ii) quantify the morphometry of the nasal conchae and nasal cavity; and (iii) develop and quantitatively support a new, clinically oriented classification of the SupNC using PCA, with the goal of improving anatomical recognition and facilitating surgical planning during endoscopic procedures.

## Material and methods

### Study and ethics

This was a descriptive, cross-sectional cadaveric study conducted on 78 head hemisections fixed in 10% neutral buffered formalin solution at the Federal University of Rio Grande do Norte (UFRN), Brazil. Of 78 initially available hemisections, 19 were excluded due to trauma, previous sinonasal surgery or poor preservation of the nasal conchae, leaving 59 head hemisections in which all nasal conchae were anatomically intact and could be clearly identified. Specimens were obtained from unclaimed or donated bodies in accordance with Brazilian regulations on the use of human cadavers for education and research. Age and sex data were not systematically available in institutional records and could therefore not be included in the analysis. In some cadavers, both hemisections from the same head were available and were treated as independent observations, which is acknowledged as a limitation of the study. The study was approved by the Institutional Research Ethics Committee (CAAE: 83959224.0.0000.5537).

Each head hemisection was catalogued, coded and photographed. The number of nasal conchae was identified, and specimens were classified as having three nasal conchae (Normal group), or a SupNC with or without an additional ZC (Variation group). SupNC were further categorized according to Orhan et al.’s [18] classification, based on their size relative to the SNC: shorter (Type A), equal (Type B) or larger (Type C).

### Measurements and classification

Using a digital calliper, a single experienced evaluator measured the following parameters three times with an accuracy of up to 0.01 mm: the distance between the anterior wall of the sphenoid sinus and the limen nasi; the vertical distance between the floor and the roof of the nasal cavity; and the anteroposterior length of the nasal conchae, defined as the distance between their anterior and posterior limits. For each nasal concha, *length* was defined as the maximum anteroposterior extent in the sagittal plane, *height* as the maximal vertical distance between its superior and inferior margins, and *area* as the two-dimensional area of its traced outline on the sagittal image.

Images of the nasal cavity were analysed using ImageJ 1.48 software (National Institutes of Health, Bethesda, MD, USA) to obtain precise morphometric data. A ruler positioned parallel to the nasal conchae was included in each image to ensure dimensional accuracy. The contours of each nasal turbinate were carefully traced using the *Freehand Selection* tool, after which the *Measure* command was applied to calculate their respective areas and heights. All measurements were performed under consistent magnification and standardized lighting conditions to minimize potential bias and ensure the reproducibility of the results. If the boundaries of a given nasal concha were not clearly defined, the corresponding morphometric parameter was not measured.

In addition to the morphometric analysis, specific morphological features were identified in the different types of the SupNC. Based on these findings, the collected data and morphological assessment of the specimens were used to propose a new classification system that integrates both morphometric and morphological aspects of the SupNC.

Principal component analysis (PCA) was applied to the morphometric data of the nasal conchae using the *prcomp* (stats package version 4.3.2) and *ggfortify* (version 0.4.17) packages in R (version 4.3.2) [24]. Variables entered into the PCA included the area, length and height of the INC, MNC, SNC and SupNC, as well as the distance between the anterior wall of the sphenoid sinus and the limen nasi and the vertical height of the nasal cavity; all variables were centred and scaled to unit variance before analysis. This technique reduces the dimensionality of the dataset by generating new variables principal components that capture the greatest proportion of variance while minimising information loss. The resulting component scores were used to visualise data clustering, after which the identified clusters were examined and compared with both Orhan et al.’s [18] classification and the classification proposed in the present study.

### Statistical analysis

GraphPad Prism® 10.0 software was used to calculate the mean and standard deviation of the data obtained from the specimens. Data normality was assessed using the Shapiro–Wilk test. For comparisons between two groups (variation vs. normal), the Student’s t-test was applied for normally distributed variables, whereas the Mann–Whitney U test was used for non-parametric distributions. For comparisons among more than two conchae within the same group, Analysis of Variance followed by the Tukey’s HSD test was applied for normally distributed data, controlling the family-wise Type I error rate in multiple comparisons. For non-parametric data, the Kruskal–Wallis test followed by Dunn’s test with correction for multiple comparisons was used. A p-value < 0.05 was considered statistically significant. Effect sizes were calculated to quantify the magnitude of differences between groups. The rank-biserial correlation coefficient (r) was used for the Mann–Whitney U test, Cohen’s d for the Student’s t-test, eta squared (*η*^2^) for the Analysis of Variance, and eta squared based on the H statistic (*η*^2^H) for the Kruskal–Wallis test.

The correlation heatmap was generated using the Python pandas library [15] (*corr()* function with “pearson” as the method) and visualised with the Seaborn (https://seaborn.pydata.org/citing.html) and Matplotlib [8] libraries.

## Results

**Fig. 1:**
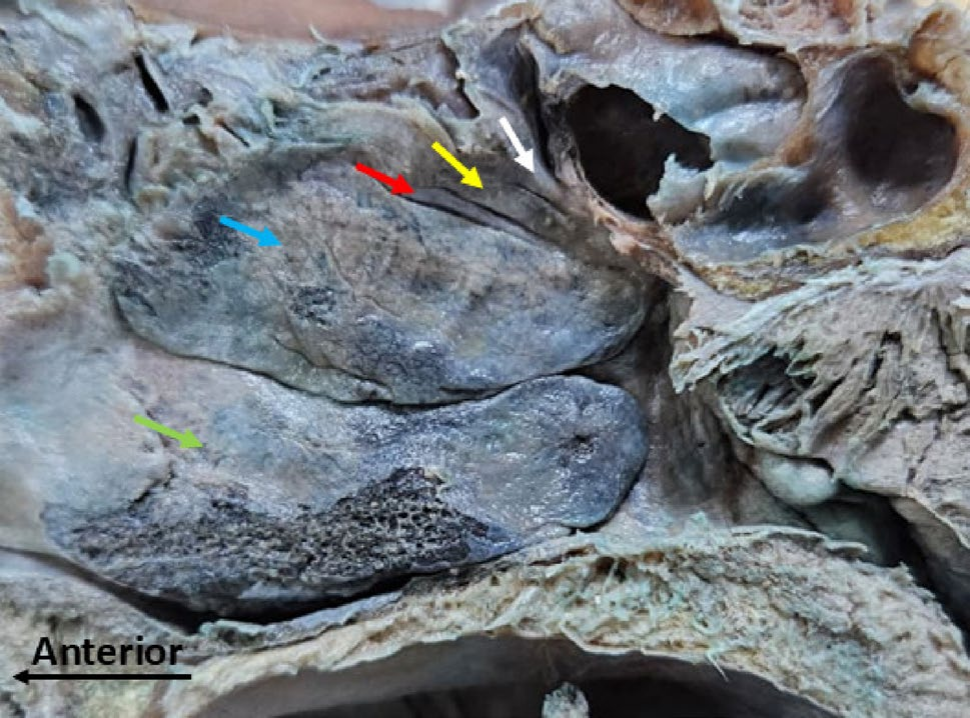
Right hemisection with the nasal cavity presenting the SupNC and the ZC. White arrow = ZC; Yellow arrow = SupNC; Red arrow = SNC; Blue arrow = MNC; green arrow = INC

Among the 59 head hemisections analysed, 37 (63%) presented three conchae, 21 (35%) presented four conchae and 1 (2%) presented five conchae. The SupNC was identified in 22/59 hemisections (37.3%), and ZC in 1/59 (1.7%) in association with a SupNC. Of the hemisections with a SupNC, 10/22 (45.5%) were on the right side and 12/22 (54.5%) on the left. The single case with ZC corresponded to a right hemisection (Fig. 1). In this specimen, both the inferior and middle nasal conchae exhibited marked hypertrophy compared with the remaining conchae.

In the Normal group, 19 hemisections (51%) were on the right side and 18 (49%) on the left. In the Variation group, 9 hemisections (43%) were right-sided and 12 (57%) left-sided. Table 1 summarizes the morphometric differences in the nasal cavity between specimens with and without the additional concha, together with their respective descriptions. The mean distance between the limen nasi and sphenoid sinus in the normal group was 54.18 ± 4.36 cm, in the variation group was 56.04 ± 4.51, the height of the nasal cavity in the normal group was 38.72 ± 5.06, in the variation group was 41.62 ± 4.94.

**Table 1:** Morphometric parameters of the normal and variation nasal conchae in the lateral nasal wall

Description	Normal area mean (SD)	Variation area mean (SD)	Normal length mean (SD)	Variation length mean (SD)	Normal height mean (SD)	Variation height mean (SD)
INC	5.64 ± 1.88	5.52 ± 2.09	46.20 ± 4.17	46.08 ± 3.91	1.19 ± 0.34	1.11 ± 0.29
MNC	3.68 ± 1.07^**^	4.45 ± 1.20	40.87 ± 3.60	41.16 ± 3.82	1.07 ± 0.21	1.17 ± 0.23
SNC	1.26 ± 0.4^**^	0.94 ± 0.36	26.13 ± 4.19^***^	21.93 ± 3.94	0.72 ± 0.24^*^	0.45 ± 0.13
SupNC	–	0.71 ± 0.31	–	14.05 ± 2.62	–	0.59 ± 0.21
ZC	–	0.44 ± 0.0	–	13.06 ± 0.0	–	0.55 ± 0.0

INC = inferior nasal concha; MNC = middle nasal concha; SNC = superior nasal concha; SupNC = supreme nasal concha; ZC = Zuckerkandl’s concha. Statistical differences between variation and non-variation groups are indicated by superscripts. * *P* < 0.0001; ** *P* < 0.02; *** *P* < 0.001;

Significant differences were observed between the normal and variation groups, with medium to large effect sizes for most parameters, including the area of the MNC (*p* < 0.02, r = 0.34), the area of the SNC (*p* < 0.02, d = 0.83), the length of the SNC (*p* < 0.001, d = 1.03), the height of the SNC (*p* < 0.0001, d = 1.38), and the height of the nasal cavity (*p* < 0.05, d = 0.58).

Statistically significant differences in morphometric parameters were identified among the nasal conchae within the same group. In the variation group, significant differences in area were observed between INC and SNC (*p* < 0.0001), INC and SupNC (*p* < 0.0001), MNC and SNC (*p* = 0.0004), and MNC and SupNC (*p* < 0.0001). The Kruskal–Wallis test indicated significant variation in area among the conchae (H(3) = 53.12, *p* < 0.0001), with a very large effect size (*η*^2^H = 0.63). In the normal group, significant differences in area were found between INC and MNC (*p* = 0.0005), INC and SNC (*p* < 0.0001), and MNC and SNC (p < 0.0001), with the Kruskal–Wallis test also revealing a very large effect size (H(2) = 67.47, *p* < 0.0001; *η*^2^H ≈ 1.00).

Significant differences in length were also detected among the nasal conchae within each group. In the variation group, differences were identified between INC and SNC (*p* < 0.0001), INC and SupNC (*p* < 0.0001), MNC and SNC (*p* = 0.0047), MNC and SupNC (*p* < 0.0001), and SNC and SupNC (*p* = 0.0399), whereas no significant difference was found between INC and MNC (*p* = 0.5050). The Kruskal–Wallis test revealed significant differences in length (H(3) = 72.61, *p* < 0.0001), with a very large effect size (*η*^2^H = 0.87). In the normal group, significant differences in length were observed between INC and MNC (*p* = 0.0027), INC and SNC (*p* < 0.0001), and MNC and SNC (*p* < 0.0001), also showing a very large effect size (H(2) = 72.45, *p* < 0.0001; *η*^2^H ≈ 1.00).

**Fig. 2:**
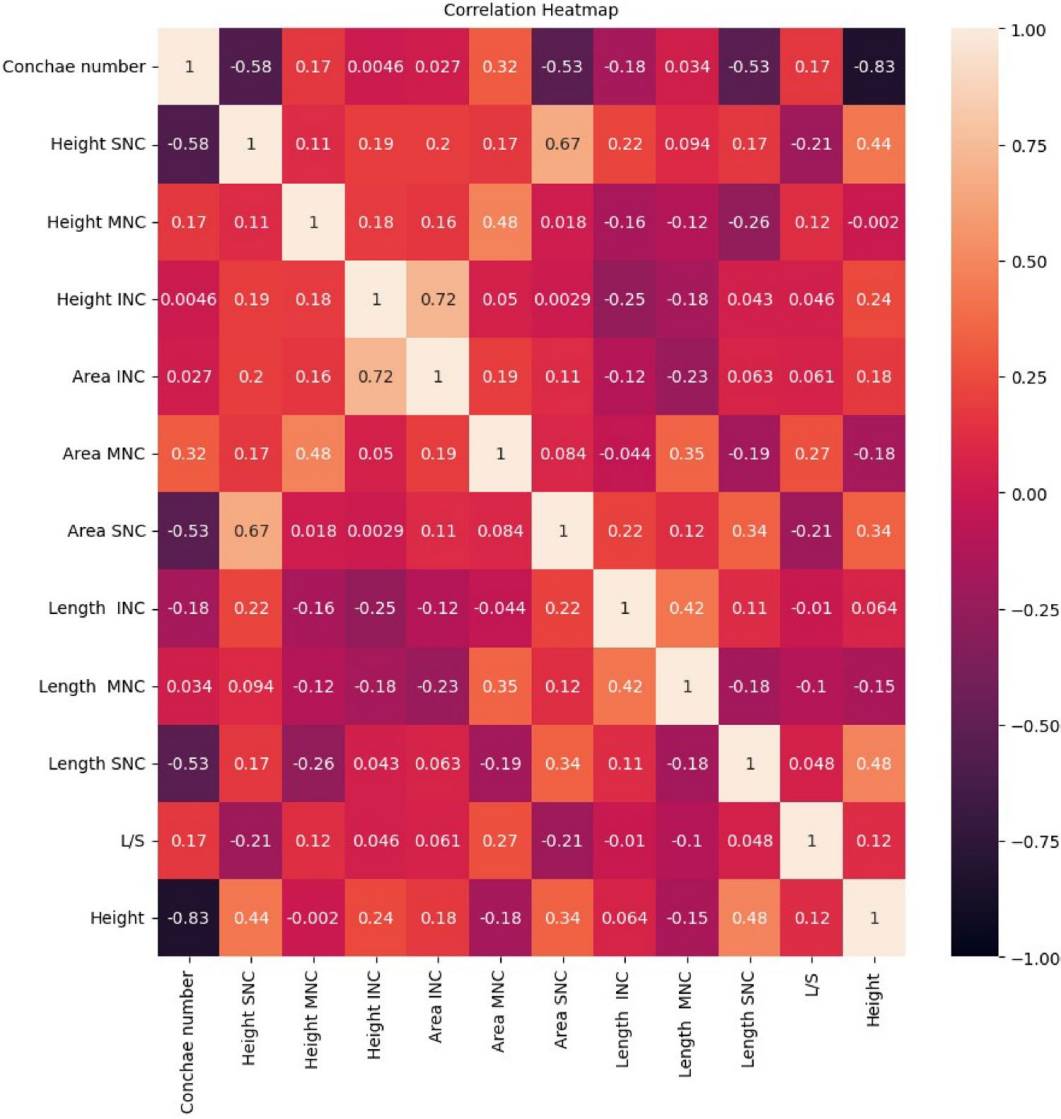
Correlation heatmap showing the correlation coefficients among the morphometric data attributes. Keys: INC = inferior nasal conchae; MNC = middle nasal conchae; SNC = superior nasal conchae; SupNC = supreme nasal conchae; L/S = distance between the limen nasi and sphenoid sinus

To evaluate the correlation coefficients among the morphometric variables, a correlation heatmap was generated (Fig. 2). The analysis revealed a strong negative correlation (r = –0.83) between the number of nasal conchae and the height of the nasal cavity, indicating an inverse relationship between these two parameters. Positive correlations were observed between the height and area of the inferior nasal concha (r = 0.72) and between the height and area of the SNC (Fig. 2). Taken together, these findings suggest that with an increasing number of nasal conchae, the vertical height of the nasal cavity tends to decrease, whereas the surface area of individual conchae, particularly the middle nasal concha, increases.

**Fig. 3:**
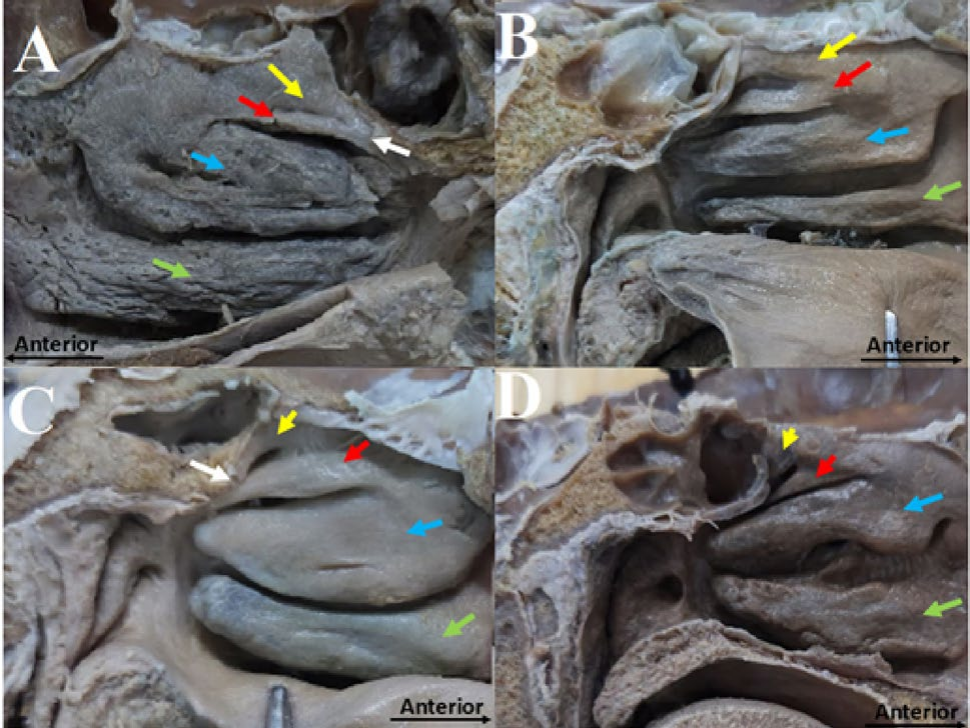
Head sagittal hemisections with representative images of the proposed classification for the SupNC described in Table 2. **A** Type Ia; **B** Type Ib; **C** Type IIa; and **D** Type IIb. White arrow = Posterior fusion between the SupNC and SNC; Yellow arrow = SupNC; Red arrow = SNC; Blue arrow = MNC; green arrow = INC

Orhan et al. [18] classified the SupNC into three types based on its length relative to the SNC: shorter (Type A), equal (Type B) or longer (Type C) (Table 2). In our material, Type B was the most frequent pattern (10/22; 45.5%), whereas Types A and C were each observed in 6/22 hemisections (27.3%). In contrast, the classification proposed in this study is based on the morphological features of the SupNC and its corresponding meatus (Table 2 and Fig. 3). In this system, Type I SupNC exhibits a straight configuration, resembling the other nasal conchae, with its meatus extending along the inferior margin. Type I is further subdivided into Type Ia, when the posterior tail of the SupNC fuses with the SNC, and Type Ib, when no such fusion occurs. Type II SupNC presents a triangular configuration, with the meatus running obliquely along its inferior border. As with Type I, Type II is subdivided into Type IIa, when the posterior tail of the SupNC joins the SNC, and Type IIb, when it remains separate. Overall, Type II (Triangular) SupNCs were more frequent than Type I (Straight) (13/22 [59.1%] vs. 9/22 [40.9%]), and posterior fusion of the SupNC with the SNC (subtypes Ia and IIa) was present in 14/22 hemisections (63.6%), whereas non-fusion (Ib and IIb) occurred in 8/22 (36.4%). These distributions are summarised in Table 2.

**Table 2:** Orhan’s et al. [18] classification *versus* the proposed classification for the SupNC

Type	Description	N (%)
Orhan’s et al. [18] classification		
A	The SupNC is shorter than the SNC	6 (27%)
B	The SupNC is equal than the SNC	10 (46%)
C	The SupNC is larger than the SNC	6 (27%)
Proposed classification		
I	The SupNC projects from the lateral nasal wall with a straight shape and a corresponding straight meatus, similar to the other conchae	9 (41%)
Ia	The posterior tail of SupNC joins the SNC	7 (32%)
Ib	The posterior tail of SupNC does not join the SNC	2 (9%)
II	The SupNC projects from the lateral nasal wall with a triangular shape, and its meatus follows an oblique course relative to the SNC	13 (59%)
IIa	The posterior tail of SupNC joins the SNC	7 (32%)
IIb	The posterior tail of SupNC does not join the SNC	6 (27%)

SNC = superior nasal concha; SupNC = supreme nasal concha; ZC = Zuckerkandl’s concha

**Fig. 4:**
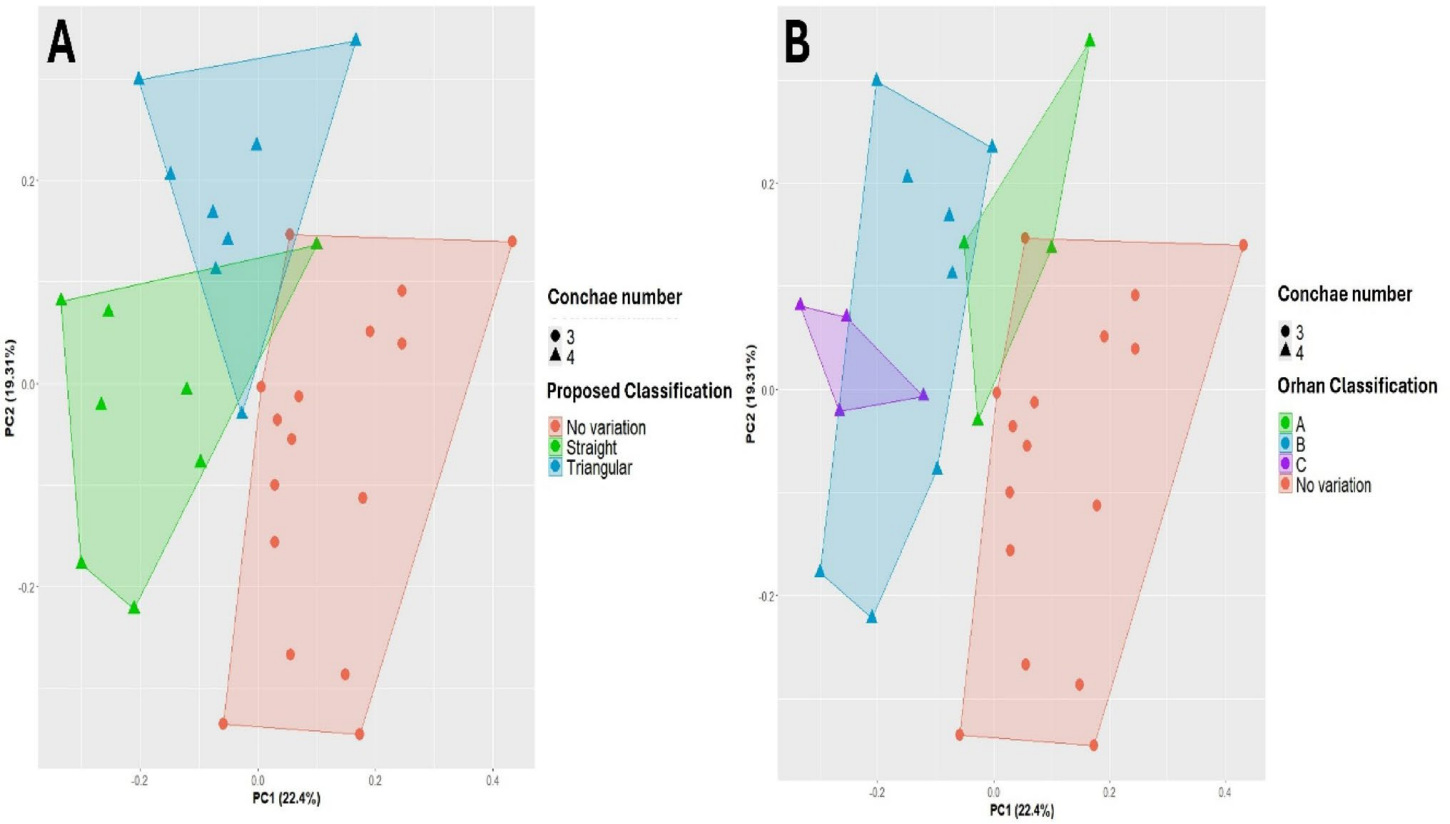
Principal Component Analysis (PCA). **A** PCA showing sample clustering according to the proposed classification and the number of conchae. **B** PCA showing sample clustering according to Orhan’s et al. [18]. classification and the number of conchae

To assess whether the proposed classification accurately reflects the morphological variation among specimens, an exploratory PCA was performed on the morphometric data. When samples were colour-coded according to the number of nasal conchae, PCA revealed two clusters, with specimens bearing three conchae clustering separately from those with four conchae. When colour-coded according to the proposed classification, a clear separation into three groups emerged: a non-variation cluster and two partially separated clusters corresponding to Straight (Type I) and Triangular (Type II) SupNCs (Fig. 4A). In contrast, when colour-coded according to Orhan et al.’s [18] length-based classification, substantial overlap particularly between Types B and C prevented a similarly distinct separation of clusters (Fig. 4B).

## Discussion

### Prevalence, morphometry, and classification

In the present study, we found a prevalence of 37.3% (22/59 hemisections) for the SupNC, broadly consistent with previous reports. Orhan et al. [18] and Gotlib et al. [6] described its unilateral or bilateral presence in up to 60 and 77% of cases, respectively. Discrepancies between cadaveric and CT-based prevalences may reflect differences in populations, imaging resolution and inclusion criteria, as well as the fact that very small bony ridges or mucosal folds on CT do not always correspond to a distinct lamella at dissection. In all specimens examined, the sphenoid ostium was located medial to the posteroinferior attachment of the SupNC and posterior to its vertical portion. Moreover, a ZC was identified in one case (1.7%), indicating that this represents a very rare anatomical variation [21].

The development of the nasal conchae begins around the eighth to tenth week of gestation, when six folds emerge within the cartilaginous nasal capsule [21]. The lowest fold, the maxilloturbinal, gives rise to the INC, whereas the remaining five folds, known as ethmoturbinals, develop into the other preturbinates [21]. From the first two or three ethmoturbinals, two to three ethmoidal turbinates arise the MNC, SNC, and, when present, the SupNC [25] which are separated by distinct grooves. By 15–16 weeks of gestation, these primitive turbinals gradually fuse into fewer (three to four) crests and become clearly differentiated, establishing the basic embryonic framework of the nasal cavity. The lateral nasal wall approaches completion by approximately 24 weeks, at which point the SNC and MNC have ossified from the ethmoid bone, while the INC develops from dual origins—the maxilla and the lateral cartilaginous capsule [17]. In this context, the complex and asynchronous morphogenesis of the nasal conchae suggests that subtle variations in ossification, fusion, or mucosal proliferation during this developmental period may contribute to the wide range of anatomical variations observed in postnatal life. Within this developmental framework, the SupNC and, more rarely, ZC can be interpreted as persistent or accentuated dorsal ethmoturbinals resulting from subtle variations in ossification, fusion or mucosal proliferation [21].

Although the regular nasal conchae frequently exhibit anatomical variations [5, 23], data on less common structures such as the SupNC and ZC remain limited. In this context, since Orhan et al.’s classification [18] does not encompass all SupNC variations identified in the present study, size criteria were complemented by the evaluation of shared morphological features. The results suggest that, although size represents an important parameter, the SupNC is more accurately defined by a combination of morphological traits. Indeed, grouping specimens according to these characteristics provides a more anatomically consistent and informative classification.

Building on Orhan’s et al. [18] framework, the proposed classification incorporates additional morphological patterns and morphometric distinctions identified in our cadaveric study. This system offers a more comprehensive categorization of the SupNC, including the configuration of the supreme meatus, thereby improving anatomical understanding, and facilitating surgical planning in the lateral nasal wall and sphenoethmoidal region.

In fact, comparison between Orhan’s et al. [18] classification and the proposed system revealed clear correspondences. SupNCs classified as Type C by Orhan et al. [18] (N = 6) corresponded mostly to Type Ia (5 of 6) in the proposed classification. Type B included a mix of Type Ia (N = 2), Ib (N = 1), IIa (N = 3), and IIb (N = 4), while Type A comprised Type Ib (N = 1), IIa (N = 3), and IIb (N = 2). These results suggest that, although size is important, SupNCs can be better represented by multiple morphological characteristics, providing a more anatomically coherent classification.

In addition, since PCA has been widely applied to biological datasets to visualise patterns and clustering among samples [12], our analysis demonstrated that the proposed classification is also reflected at the data-clustering level: specimens formed three clusters, corresponding to a non-variation group and two partially separated clusters representing Straight (Type I) and Triangular (Type II) SupNCs. Because PCA groups samples based on quantitative data, it provides a more robust support for the classification than purely visual or arbitrary criteria. In contrast, Orhan’s classification [18] did not show clear separation between groups in the PCA, with considerable overlap, particularly between Types B and C suggesting that length-based categories alone may not align with the underlying morphometric structure.

### Clinical standpoints

A strong negative correlation was observed between the number of nasal conchae and the height of the nasal cavity, indicating that the presence of additional conchae may be associated with a reduction in the vertical dimension of the cavity. This finding may indicate a compensatory spatial adjustment within the nasal cavity, where the development of an extra concha could limit overall height to maintain proportionality and airflow dynamics. Conversely, the positive correlations between concha height and area both in the INC and SNC suggest that larger conchae tend to expand proportionally in multiple dimensions, which may enhance their surface area for air conditioning and filtration. Together, these relationships highlight an apparent balance between structural complexity and functional efficiency within the nasal cavity.

From a clinical perspective, anatomical variations of the lateral nasal wall such as concha bullosa [11], the presence of accessory nasal conchae [4], or hypertrophy of the INC [17]— as well as variations in other regional structures, can contribute to chronic sinusitis, nasal cavity stenosis, and may be associated with certain types of tumors. These variations can also affect neighboring structures, potentially compromising them due to altered anatomy [26]. In addition, the presence of the SupNC, along with the MNC and SNC and the orbital floor, can be considered an additional surgical landmark in endoscopic sinus and skull base surgeries—particularly in cases of inflammatory polyposis or sinonasal tumors, where excessive bleeding and distortion of the normal anatomy are common [1]. For instance, in a previous report, sphenoidotomy was performed with the SupNC serving as an anatomical landmark, and because the sphenoid sinus ostium is consistently located just medial to its inferior portion, dissection was directed medial to this segment to safely access the sphenoid sinus [3].

In challenging functional endoscopic sinus surgery cases [2] with distorted anatomy of the middle and superior nasal conchae, identifying the SupNC may provide an additional orientation point towards the sphenoid ostium and sphenoethmoidal recess. A triangular (Type II) SupNC with an oblique supreme meatus may redirect the surgical trajectory and modify the apparent course of the supreme and superior meatuses, whereas a straight SupNC fused posteriorly to the SNC (Types Ia and IIa) may partially conceal a narrow sphenoethmoidal recess or a high-riding sphenoid ostium. Additionally, it remains unclear whether the straight (Type I) and triangular (Type II) configurations of the SupNC present distinct anatomical and clinical characteristics, particularly regarding meatal orientation and surgical access. Clarifying these differences may facilitate their incorporation into preoperative CT assessment and help surgeons anticipate difficult access routes and choose safer angles of approach.

Most previous studies have relied on imaging data, particularly CT, which remains the gold standard for assessing nasal anatomy and identifying anatomical variations [6]. Nevertheless, cadaveric studies offer a more accurate and detailed method for confirming these variations and performing morphometric measurements. Thus, direct observation and dissection enable the accurate identification of fine structures that may be overlooked or poorly visualized on imaging, thereby complementing radiological findings and contributing to a more comprehensive understanding of sinonasal anatomy.

It is important to acknowledge certain limitations of the present study. First, fixation in 10% neutral buffered formalin may have caused minor, non-uniform changes in morphometric parameters. all morphometric measurements were performed by a single observer, which may have introduced observer-related bias. Moreover, the sample size was relatively modest (59 head hemisections) [9], and age and sex data were not systematically available and could therefore not be analyzed, preventing the assessment of potential demographic influences on SupNC morphology and prevalence. Additionally, some cadavers contributed bilateral hemisections that were treated as independent observations, introducing a degree of clustering that may slightly affect variance estimates. Finally, we did not have access to pre-mortem CT scans for these donors, so direct radiological–anatomical correlations could not be performed. Nevertheless, while these factors represent constraints, our results provide valuable insights into the prevalence and morphometry of the SupNC. Future investigations with larger samples are warranted to confirm and expand upon these findings.

Overall, a comprehensive understanding of both typical anatomy and its possible variations is crucial for sinus surgeons. Awareness of such variations and classifications, supported by modern imaging modalities, may facilitate more accurate preoperative planning and help prevent intraoperative complications or unexpected anatomical challenges. However, the clinical implications of the present findings remain partly speculative, and the proposed classification requires external or radiological validation, which should be addressed in future studies.

## Conclusion

By combining gross anatomy, detailed morphometry and PCA-based clustering, this study proposes a reproducible, clinically oriented classification of the supreme nasal concha. The framework distinguishes Straight (Type I) and Triangular (Type II) SupNCs, with or without posterior fusion to the superior nasal concha, and may be applicable in anatomical, radiological and endoscopic practice to improve preoperative assessment and intraoperative orientation in the sphenoethmoidal region.

## Data Availability

The data that support the findings of this study are available from the corresponding author upon reasonable request.
